# 1165. Longitudinal Evaluation of SARS-CoV-2 Antibody Response Using Dried Blood Spot Samples Following Vaccination with Three and Four Doses of mRNA-1273, BNT162b2 and/or ChAdOx1-S in Adults Aged 50 and Above: Interim Analysis from the PREVENT-COVID Study

**DOI:** 10.1093/ofid/ofad500.1005

**Published:** 2023-11-27

**Authors:** Brynn McMillan, Gabrielle N Gaultier, Ana Citlali Marquez, Tahereh Valadbeigy, Bonny So, Sydney Schwartz, Hennady Shulha, Mandy Lo, Bing Cai, Muhammad Morshed, Inna Sekirov, Karen Simmons, Sofia R Bartlett, Megan K Levings, Theodore Steiner, James Zlosnik, Danuta Skowronski, Agatha N Jassem, Manish Sadarangani

**Affiliations:** University of British Columbia, Vancouver, British Columbia, Canada; University of British Columbia, Vancouver, British Columbia, Canada; BC Centre for Disease Control, Vancouver, British Columbia, Canada; UBC, Burnaby, British Columbia, Canada; University of British Columbia, Vancouver, British Columbia, Canada; University of British Columbia, Vancouver, British Columbia, Canada; Vaccine Evaluation Center, BC Children's Hospital Research Institute, The University of British Columbia, Vancouver, British Columbia, Canada; University of British Columbia, Vancouver, British Columbia, Canada; University of British Columbia, Vancouver, British Columbia, Canada; University of British Columbia, Vancouver, British Columbia, Canada; University of British Columbia, Vancouver, British Columbia, Canada; University of British Columbia, Vancouver, British Columbia, Canada; BC Centre for Disease Control, Vancouver, British Columbia, Canada; UBC, Burnaby, British Columbia, Canada; University of British Columbia, Vancouver, British Columbia, Canada; BC Centre for Disease Control/ University of British Columbia Department of Pathology and Laboratory Medicine, Vancouver, British Columbia, Canada; BC Centre for Disease Control, Vancouver, British Columbia, Canada; University of British Columbia, Vancouver, British Columbia, Canada; University of British Columbia, Vancouver, British Columbia, Canada

## Abstract

**Background:**

Multiple combinations of COVID-19 vaccine regimens have been used in Canada throughout the SARS-CoV-2 immunization campaign. Studies evaluating the humoral immune response following COVID-19 vaccination in community dwelling older adults remain limited. This study assessed COVID-19 vaccine elicited antibody responses in older adult populations, alongside factors that influence antibody responses.

**Methods:**

Community dwelling adults aged 50 to 87 years (mean=65) were enrolled (n=612). Detection of index SARS-CoV-2 anti-spike IgG (anti-S-IgG) concentration and surrogate neutralization were performed on dried blood spot samples via two multiplex assays (Meso Scale Diagnostics). Anti-S-IgG concentration and surrogate neutralization were quantified following mRNA (mRNA-1273 [m-1273], BNT162b2 [BNT]) or viral vector (ChAdOx1-S [ChAd]) vaccination. Vaccine groups were compared using one-way ANOVA and Tukey-Kramer multiple comparisons tests. Multivariable regression analyses evaluated influences of demographic and clinical factors on humoral immune responses.

**Results:**

Three doses of m-1273 resulted in significantly higher anti-S-IgG compared with three BNT doses at four months (geometric mean concentration; 10167 AU/mL vs. 5412 AU/mL, *P*=0.009) post dose three. Three dose mixed vaccination with ChAd, m-1273 and BNT resulted in comparable anti-S-IgG concentration to three dose m-1273 at four months post dose three. Three doses of either m-1273 or mixed mRNA containing vaccines was associated with significantly higher surrogate neutralization compared with three BNT doses at four months (46% & 43% vs. 34%, *P*=0.002) post dose three. No significant difference in anti-S-IgG concentration was observed in four dose vaccination regimens. SARS-CoV-2 infection, health status of excellent or very good, and m-1273 containing vaccine regimens positively influenced the antibody response.

**Conclusion:**

Immunization schedules including a minimum of one m-1273 dose elicited the strongest and most durable antibody responses compared with BNT only containing regimens. There is no established correlate of protection for COVID-19, and as such this data should be interpreted alongside vaccine effectiveness studies. Omicron and XBB specific antibody responses will be compared.

**Disclosures:**

**Sofia R. Bartlett, PhD**, Abbvie: Advisor/Consultant|Abbvie: Grant/Research Support|Cepheid: Advisor/Consultant|Gilead: Advisor/Consultant|Gilead: Grant/Research Support **Theodore Steiner, MD, FRCPC**, Edesa: Grant/Research Support|Ferring: Advisor/Consultant|Ferring: Grant/Research Support|Qu Biologics: Advisor/Consultant|Qu Biologics: Stocks/Bonds|Seres: Grant/Research Support **Manish Sadarangani, BM BCh, FRCPC, DPhil**, GlaxoSmithKline: Grant/Research Support|Merck: Grant/Research Support|Moderna: Grant/Research Support|Pfizer: Grant/Research Support|Sanofi Pasteur: Grant/Research Support|Seqirus: Grant/Research Support|Symvivo: Grant/Research Support|VBI Vaccines: Grant/Research Support

